# Everyday Ageism and Health: Evidence From the National Poll on Healthy Aging

**DOI:** 10.1093/geroni/igaa057.2558

**Published:** 2020-12-16

**Authors:** Julie Ober Allen, Erica Solway, Matthias Kirch, Dianne Singer, Jeffrey Kullgren, Preeti Malani

**Affiliations:** 1 University of Oklahoma, Ann Arbor, Michigan, United States; 2 University of Michigan, Ann Arbor, Michigan, United States; 3 University of Michigan Medical School, Ann Arbor, Michigan, United States

## Abstract

This study examined the prevalence of everyday ageism, routine types of age-based discrimination, prejudice, and stereotyping that older adults encounter in their daily lives, and its relationships with health in a nationally representative sample age 50-80 (N=2,048, 52% female, 71% White). Nearly all older adults said they sometimes or often experienced everyday ageism (96% age 65-80, 92% age 50-64). The most common types were beliefs that health problems were an inevitable part of getting older (78%), hearing jokes about aging/older people (61%), and seeing material suggesting that older adults were unattractive/undesirable (38%). Those reporting more experiences with everyday ageism (>3 types) were less likely than those reporting fewer types to have excellent/very good physical health (31% vs. 50%); similar results were found for mental health (60% vs. 80%). This poll documented the ubiquity of minor, but not inconsequential, everyday ageism reported by older adults and its potential ramifications for health.

